# Safety and efficacy of precision hepatectomy in the treatment of primary liver cancer

**DOI:** 10.1186/s12893-023-02148-7

**Published:** 2023-08-17

**Authors:** Junhong Zhang, Pengfei Zhang, Jinglin Cao

**Affiliations:** Department of Hepatobiliary Surgery, The Third Hospital of Hebei Medical University, No. 139 Zi Qiang Road, Shijiazhuang, 050000 Hebei Province China

**Keywords:** Precision hepatectomy, Conventional hepatectomy, Primary liver cancer

## Abstract

**Background:**

The aim of this study was to investigate the safety and efficacy of precision hepatectomy in the treatment of primary liver cancer.

**Methods:**

An randomized controlled trial of 98 patients with primary liver cancer admitted to our hospital from February 2020 to February 2021 were analyzed for the study, and they were divided into 49 cases each in the control group (conventional hepatectomy) and the study group (precision hepatectomy) according to the different surgical methods. The surgical condition, complications and follow-up results of the two groups were counted, and the liver function and immune function of the two groups were observed before and 1 week after surgery.

**Results:**

The operation time, intraoperative bleeding, hospitalization time and anal venting time in the study group were less than those in the control group (P < 0.05). One week after surgery, AST, TBiL, ALT and ALB levels decreased, with in the study group significantly higher than those in the control group (P < 0.05); CD4+, CD3 + and CD4+/CD8 + levels were significantly higher in the study group (P < 0.05). The incidence of complications in the study group was significantly lower than that in the control group (P < 0.05). After 2 years of follow-up, the recurrence rate and mortality rate of the study group were lower than those of the control group (P < 0.05); the difference was not statistically significant when comparing the metastasis rate between the two groups (P > 0.05).

**Conclusion:**

Precision hepatectomy can effectively treat primary liver cancer with high safety and could be promoted in clinical practice.

## Background

Primary liver cancer refers to malignant tumors occurring in hepatocytes or intrahepatic bile duct cells, and is one of the most common malignancies in clinical practice, ranking fifth in incidence among malignancies worldwide and the third leading cause of cancer-related deaths [1–3]. The annual number of new cases of primary liver cancer in China accounts for more than 50.0% of the worldwide incidence, and the survival rate within 5 years is 26-50%, with a tumor-free survival rate of only 13-29%, and relatively high postoperative complications and mortality rates, which have a serious impact on patients’ quality of life and life health [2]. With the increasing number of primary liver cancer patients worldwide, the optimization of treatment options for primary liver cancer has become a hot topic of clinical research. At present, the principle of clinical treatment for primary liver cancer is to provide targeted treatment according to its different stages, and surgery is still the main treatment method [4, 5].

How to improve the resection rate of primary liver cancer remains a hot topic of clinical research. Precision hepatectomy is a new concept and technical system for the treatment of liver cancer, pursuing the ideal goal of minimizing invasion and maximizing liver protection and obtaining the best recovery outcome, and accurate assessment of the liver before surgery is particularly important [6, 7]. In recent years, 3D reconstruction techniques have been increasingly used in primary liver cancer patients, but there are few clinical studies on precision hepatectomy guided by 3D reconstruction techniques in primary liver cancer [8]. Based on this, this study will investigate the safety and efficacy of precision hepatectomy in the treatment of primary hepatocellular carcinoma.

## Materials and methods

### General data

98 patients with primary liver cancer admitted to our hospital from February 2020 to February 2021 were analyzed, and they were divided into 49 cases each in the control group (conventional hepatectomy) and the study group (precision hepatectomy) according to the surgical method. The study protocol complied with the relevant requirements of the World Medical Association Declaration of Helsinki [9]. The study protocol was approved by the institutional review board of The Third Hospital of Hebei Medical University (EIC21-39, date: 2021-05-06). The patients who participated provided informed consent.

### Inclusion and exclusion criteria

Inclusion criteria: according to the diagnostic criteria of the “Code of Practice for the Treatment of Primary Liver Cancer (2017 edition)” [10]; pathological skin examination of surgically resected specimens confirmed as primary liver cancer; no previous history of upper abdominal surgery and hepatobiliary system diseases; normal function of important organs such as heart, brain, lung and kidney; no distant organ metastases detected before surgery; lesions did not invade the first and second hilar regions; no severe hyperglycemia, increased blood pressure and coagulation before surgery abnormalities.

Exclusion criteria: mental illness; metastatic liver cancer; severe co-morbidities; history of previous upper abdominal surgery, especially hepatobiliary surgery; total hepatic occupying lesions, or severe cirrhosis that cannot be surgically resected or is not surgically possible; preoperative detection of distant organ metastases; survival time < 3 months.

### Methods

The control group was given conventional surgical treatment with general anesthesia by tracheal intubation, and the surgical position was supine, with routine disinfection and surgical towel. The tumor tissue was completely exposed within the field of vision, the first and second hepatic hilum was blocked, the large blood vessels and the hepatic hilum were clamped, the peripheral ligaments such as the hepatic round ligament and the sickle ligament were separated, and resection was performed at a position about 2 cm from the edge of the tumor tissue, from superficial to deep, from inside to outside. The residual blood vessels and bile ducts are ligated or sutured. The wound is rinsed and observed for biliary fistulas and bleeding, followed by hemostasis, placement of an abdominal drain and closure of the abdomen.

The study group was given precision hepatectomy.

(1) CT examination: A 64-row spiral CT machine from GE, USA, with a layer thickness of 1.25-5 mm, scanned from the top of the diaphragm to the lower edge of the liver, and divided into a plain phase, an arterial phase and a portal phase. Patient CT data is collected and stored in Digital Image in Medicine (DICOM) format for data transfer via PACS system or data retention on disk.

(2) Three-dimensional reconstruction of the liver: The CT data of all patients are obtained in the IQQA-Liver, a three-dimensional liver reconstruction software produced by EDDA, USA. The system automatically extracts the image information of the liver parenchyma, lesions, portal veins and parts of the hepatic veins, reconstructs the spatial structure of the liver parenchyma and the intrahepatic ducts, segments the intrahepatic lesions, and the three-dimensional reconstruction shows their spatial shape, size, parts and the interrelationship between them and the intrahepatic ducts. The 3D reconstruction shows the spatial shape, size, parts and interrelationships with the intrahepatic ducts. The 3D and 2D fusion tools included in the software are used to check the authenticity of the image, to dynamically observe the reconstructed model in the 3D plane, and to manually outline and modify the few reconstruction discrepancies to remove irrelevant parts. For patients with intrahepatic bile duct dilatation, the bile ducts were identified, extracted and reconstructed, while the posterior inferior vena cava was completely reconstructed by hand. The model is rotated in the 3D plane to view the lesion from multiple angles, and the software automatically generates an image report of all the data processed by the reconstruction and saves the dynamic data in AVI video format using the video recording function. (3) Liver volume measurement: In the three-dimensional liver reconstruction system, the whole liver volume, lesion volume, pre-resection liver volume and remaining liver volume of the virtual surgical model, the distance between the lesion and each duct, the internal diameter of each duct in the section, and other parameters were measured in real time using the measurement tools brought by the system, and the measured data were generated and saved as text reports. The whole liver volume, focal volume, pre-operative pre-resected liver volume and remaining liver volume were also measured in 49 patients using our PACS system to obtain two-dimensional measurements of liver volume in all cases.

(4) Virtual surgical design: The 3D reconstructed model of the liver of 49 patients was observed from multiple angles in 3D space, the relationship between the lesion and the hepatic vein, portal vein and bile duct was clarified, the invasion of the first and second hepatic hilum and the posterior inferior vena cava was analyzed, the tumor resection boundary was set by applying the tumor safety boundary range tool with reference to each measurement data, and finally the 3D reconstructed model was simulated to be cut by using the software liver space cutting tool. The patient’s liver volume and the percentage of the whole liver were measured in real time, and all resection options were analyzed and compared to assess whether the volume of the remaining liver could meet the postoperative compensations according to the patient’s lesion volume and the invasion of important structures in the adjacent liver. The design of the individualized virtual surgical resection is completed, and different surgical options are compared, selected and optimized to finalize the surgical plan and guide the clinical procedure.

(5)Tracheal intubation general anesthesia, take the supine position, the right subcostal incision into the abdomen, first explore and then use the B-ultrasound and preoperative 3D virtual planning to determine the pre-cut line, selectively block the hepatic artery and portal vein branches of the liver segment to which the tumor belongs, and cut off the liver parenchyma along the boundary of the liver segment, as far as possible to ensure complete resection of the tumor while reducing the damage to its surrounding vascular structure, free the tumor tissue along the ischemic line by ultrasonic scalpel combined with clamp method, ligate or suture the broken blood vessels and bile ducts, confirm the section to stop bleeding, and use argon to spray the section.

### Observation items

Statistics on the surgical situation, complications and follow-up results of the two groups. Observation of liver function and immune function before and 1 week after surgery in both groups.

Surgery: Comparison of the time of surgery, intraoperative bleeding, length of hospital stay and time of anal venting between the two groups.

Liver function: Venous blood was drawn from patients before and 1 week after surgery, and Aspartate aminotransferase (AST), total bilirubin (TBiL), alanine transaminase (ALT) and albumin (ALB) were measured using an AU5800 automatic biochemistry instrument manufactured by American Beckman Coulter.

Immune function: 5ml of venous blood was drawn from patients before and 1 week after surgery, centrifuged at 3000r/min (10 min) and sent for examination of induced T cells (CD4+), mature T lymphocytes (CD3+) and CD4+/suppressor T cells (CD4+/CD8+).

Complications: The occurrence of complications such as pleural effusion, bile leak, abdominal infection and lung infection were counted in both groups.

Follow-up: Patients were followed up at 6th month, 12th months, 2nd and 3rd year after surgery by telephone, WeChat or home visits after surgery. Consult the patient for symptoms such as abdominal pain, jaundice, gastrointestinal symptoms, etc. If present, the patient should go to the clinic for relevant investigations, in combination with clinical symptoms along with AFP levels and PET-CT results to determine recurrence or metastasis.

### Statistical analysis

SPSS 21.0 software was used for statistical analysis. If the measurement data conforms to the normal distribution, the mean ± standard deviation is used to describe it, and the t test is used. If it does not conform to the normal distribution, the rank sum test is used. The count data were described by frequency (composition ratio). The one-way ordered data were compared by rank sum test, and the disordered data were tested by χ2 test. P < 0.05 was considered statistically significant.

## Results

### Comparison of general data between the two groups

Study group: 29 males and 20 females, aged 32–73 years, mean age (56.29 ± 12.49); tumor diameter was 4–11 cm, mean tumor diameter (7.65 ± 2.03) cm. Control group: 26 males and 23 females, aged 31–72 years, mean age (56.17 ± 12.56); tumor diameter was 4–11 cm, mean tumor diameter (7.59 ± 2.01) cm. No statistical difference was observed in general data of the two groups (P > 0.05), as shown in Table 1.

**Table 1:** Comparison of general data between the two groups

Indicators	Study group(49)	Control group(49)	t	P
Age (years)	56.29 ± 12.49	56.17 ± 12.56	0.047	0.963
Sex [M/F (n)	29/20	26/23		
Cirrhosis			0.368	0.544
Yes	25	22		
No	24	27		
Tumor diameter (cm)	7.65 ± 2.03	7.59 ± 2.01	0.147	0.881
Chlid-Pogh grading (n)			0.272	0.602
Grade A	39	41		
Grade B	10	8		
TNM stage (n)			0.511	0.475
Stage I	36	39		
Stage II	13	10		
CNLC staging (n)			0.047	0.828
Stage I	33	34		
Stage II	16	15		
Pathological type (n)			0.299	0.585
Hepatocellular carcinoma	40	42		
Bile duct cell carcinoma	9	7		
Cirrhosis (n)				
Comorbidities (n)				
Hypertension	6	9	0.708	0.419
Stroke	8	6	0.333	0.564
Coronary heart disease	7	5	0.381	0.538

### Comparison of surgical conditions between the two groups

The operation time, intraoperative bleeding, hospital stay and time of anal venting were less in the study group than in the control group with significant differences (P < 0.05), as shown in Table 2.

**Table 2:** Comparison of surgery between the two groups

Indicators	Study group(49)	Control group(49)	t	P
Operating time (min)	91.29 ± 7.91	98.47 ± 8.27	-4.392	<0.001
Intraoperative bleeding (mL)	220.71 ± 10.23	248.91 ± 12.33	-12.321	<0.001
Anal venting time (h)	72.19 ± 7.37	81.23 ± 7.48	-6.026	<0.001
Length of hospital stay (days)	9.27 ± 1.27	11.38 ± 1.32	-8.063	<0.001

### Comparison of liver function between the two groups

Before surgery, there was no statistically significant difference between the levels of AST, TBiL, ALT and ALB in the two groups (P > 0.05); 1 week after surgery, the levels of AST, TBiL, ALT and ALB decreased in both groups, with the levels of AST, TBiL, ALT and ALB in the study group markedly higher than those in the control group(P < 0.05), as shown in Table 3.

**Table 3:** Comparison of liver function between the two groups

Indicators	Time	Study group(49)	Control group(49)	t	P
AST(U/L)	Before surgery	58.92 ± 7.76	58.68 ± 7.89	0.152	0.881
	1 week after surgery	41.38 ± 6.37	34.98 ± 6.74	4.831	<0.001
TBiL(U/L)	Before surgery	31.87 ± 5.91	32.09 ± 5.65	-0.188	0.851
	1 week after surgery	17.47 ± 5.12	14.39 ± 5.09	2.986	0.002
ALT(U/L)	Before surgery	46.76 ± 4.92	45.69 ± 5.01	1.067	0.289
	1 week after surgery	32.81 ± 5.37	26.98 ± 5.48	5.319	<0.001
ALB(U/L)	Before surgery	38.71 ± 5.81	38.65 ± 6.09	0.051	0.961
	1 week after surgery	55.39 ± 6.01	46.19 ± 6.06	7.546	<0.001

### Comparison of immune function between the two groups

Before surgery, there was no difference in CD4+, CD3 + and CD4+/CD8 + levels between the two groups (P > 0.05); 1 week after surgery, CD4+, CD3 + and CD4+/CD8 + levels decreased in both groups, and CD4+, CD3 + and CD4+/CD8 + levels in the study group were significantly higher than those in the control group (P < 0.05), see Table 4.

**Table 4:** Comparison of immune function between the two groups

Indicators	Time	Study group(49)	Controlgroup(49)	t	P
CD4^+^(%)	Before surgery	34.98 ± 3.87	35.01 ± 4.01	-0.038	0.971
	1 week after surgery	27.02 ± 3.93	20.87 ± 3.87	7.792	<0.001
CD3^+^(%)	Before surgery	62.09 ± 4.09	62.17 ± 3.16	-0.108	0.914
	1 week after surgery	48.76 ± 4.21	53.29 ± 3.76	-5.685	<0.001
CD4^+^/CD8^+^(%)	Before surgery	1.31 ± 0.19	1.33 ± 0.21	-0.494	0.622
	1 week after surgery	0.93 ± 0.16	0.64 ± 0.17	-291.151	<0.001

### Comparison of the incidence of complications between the two groups

We recorded the incidence of pleural effusion, bile leak, abdominal infection, pulmonary infection and total complications in both groups and found that the incidence of complications in the study group was significantly lower than that in the control group, with a significant difference (P < 0.05), as shown in Table 5.

**Table 5:** Comparison of the incidence of complications between the two groups

Indicators	Study group(49)	Control group(49)	χ^2^	P
Pleural effusion	1	3		
Biliary Leakage	0	2		
Abdominal Infection	0	1		
Pulmonary infection	0	1		
Total incidence (%)	1(2.04%)	7(14.29%)	4.901	0.027

### Comparison of the follow-up results between the two groups

After 2 years of follow-up, the recurrence rate and mortality rate of the study group were lower than those of the control group, with significant differences (P < 0.05); there was no significant difference in the metastasis rate between the two groups (P > 0.05), as shown in Table 6.

**Table 6:** Comparison of the incidence of complications between the two groups

Indicators	Study group(49)	Control group(49)	χ^2^	P
Relapse	1	7	4.900	0.027
Metastasis	1	4	1.897	0.168
Death	1	7	4.900	0.027

## Discussion

Primary liver cancer includes three types: mixed liver cancer, cholangiocarcinoma and hepatocellular carcinoma [11, 12]. At present, the etiology of primary liver cancer is not completely clear. Studies have pointed out that hepatitis B virus, alcohol, cirrhosis, hepatitis C virus infection and so on can induce liver cancer [13–16]. Early-stage primary liver cancer lacks typical clinical symptoms and has been overlooked by patients, yet as the disease progresses, it increases the difficulty of treatment and has an impact on the prognosis level of patients [17]. At present, surgical resection is the main treatment for primary liver cancer, which can effectively remove tumor tissue and improve patients’ condition, but this procedure is more traumatic to patients’ body and patients will induce a series of complications after surgery, which reduces patients’ quality of life [18, 19]. With the continuous advancement in liver surgery and the rapid development of imaging and improved methods of assessing liver reserve function in the perioperative period, precision hepatocellular carcinoma removal has gained widespread clinical attention as a new surgical concept and technical system with the ultimate goal of minimizing trauma, maximining liver protection and achieving the most desirable recovery outcome [20, 21].

Precise surgical operation means that on the basis of precise pre-operative assessment and sophisticated surgical planning, the operation is carried out according to a previously drawn up plan, with a delicate operation that presupposes radical resection, grasps the concept of minimal invasion, minimizes unnecessary tissue damage and applies advanced techniques to control intraoperative bleeding [6, 22]. Especially for complex liver resections. Due to certain technical bottlenecks in the image post-processing workstation itself, which affect the preoperative assessment of liver surgery by imaging techniques [23]. The reconstructions done on the basis of medical imaging technology workstations are not true fully quantified 3D reconstructions, which can be done by simple measurements but not by arbitrary reconstruction of 3D objects [24]. The development of modern surgical concepts, the higher requirements of liver surgeons for the diagnosis and treatment of diseases and advanced computer virtual technology have contributed to the development of digital medicine. A variety of digital liver 3D reconstruction systems have emerged in liver surgery, which rapidly complete 3D liver reconstruction based on traditional imaging data and individualized quantitative analysis of liver data, while enabling virtual surgery [25]. There are the earliest foreign reports of 3D liver reconstruction, the development of assisted surgery systems and their clinical use. Since 2013, we have acquired the EDDA liver 3D reconstruction surgical planning system [26] from the US and performed precision hepatectomy under the guidance of this system, with the aim of observing the value of precision hepatectomy in patients with primary liver cancer.

Deng et al. showed that precision hepatectomy was effective in treating elderly patients with primary liver cancer, improving immune function and having a high safety profile [27]. The study by Luo et al. pointed out that precision hepatectomy could effectively reduce the amount of less intraoperative bleeding, lower postoperative complication rate, shorter hospital stay and high tumor-free survival rate in patients with primary liver cancer [28]. The results of our study showed that the operative time, intraoperative bleeding, hospitalization time and time of anal venting in the study group were all less than those in the control group, which is consistent with the above-mentioned findings, suggesting that precision hepatectomy can effectively promote patients’ recovery.

AST, TBiL, ALT and ALB are the main clinical indicators of liver damage, of which the higher the expression of ALT enzyme activity, it is likely that hepatocyte necrosis is present [29]. AST is mainly found in mitochondria, and when high AST levels are detected, excluding cardiomyopathy, it confirms massive necrosis in the mitochondria of the liver. ALB is the most abundant protein in the plasma of vertebrates [30]. Its role in binding and transporting endogenous and exogenous substances, maintaining blood colloid osmotic pressure, scavenging free radicals, mechanism of platelet aggregation and anticoagulant function is of great importance in the life course. TBiL is an important indicator of the patient’s liver function, which can guide treatment and reflect the prognosis of the tumor [31]. Duan et al. showed that the clinical treatment of primary hepatocellular carcinoma can be performed by precision hepatectomy, which has better recovery of residual liver function and more stable immune factors than conventional hepatectomy, resulting in better outcomes [32]. The results of our study showed that the levels of AST, TBiL, ALT and ALB decreased in both groups 1 week after surgery, with the levels of AST, TBiL, ALT and ALB in the study group being significantly higher than those in the control group (P < 0.05), suggesting that precision hepatectomy can effectively reduce the damage to liver function.

T-lymphocyte subsets provide accurate feedback on the immune function of the body and are an indicator of whether the body’s immune function is in balance, with CD3 + being the main active cell and CD4 + being the helper T-lymphocyte [33]. Decreasing in CD4+/CD8 + levels indicates a more severe impairment of the body’s immune function, while a lower immune function is associated with a more severe trauma [34]. The results of our study revealed that the CD4+, CD3 + and CD4+/CD8 + levels decreased in both groups 1 week after surgery, suggesting that precision hepatectomy has less impact on immune function. The study by Tang et al. [35]also showed that partial hepatectomy for primary hepatocellular carcinoma was less invasive, had faster postoperative recovery, and had less impact on cellular immune function, which is consistent with the results of our study.

There are shortcomings in the study, as 3D liver reconstruction is a preoperative virtual surgical operation that can only rely on preoperative scanned images, and it is difficult to maintain consistency between preoperative imaging data and intraoperative completion of the situation, as it is difficult to avoid degeneration of the liver tissue due to traction during surgery for primary liver cancer, and the process does not allow for real-time images to guide the surgery, so further research is needed on how to make 3D reconstruction techniques better for use in surgery.

## Conclusion

In summary, precision hepatectomy can effectively shorten the operative time, hospital stay and anal venting time of primary liver cancer patients, reduce intraoperative bleeding, mitigate damage to liver function, have less impact on immune function and have fewer complications. In addition, precision hepatectomy can effectively reduce recurrence and mortality rates, facilitate patient recovery and improve patient prognosis.

## Data Availability

The datasets used during the current study are available from the corresponding author on reasonable request.

## Abbreviations

ALB: albumin (ALB)
ALT: alanine transaminase
AST: Aspartate aminotransferase
TBiL: total bilirubin
